# Flavonoids from *Pterogyne nitens* Inhibit Hepatitis C Virus Entry

**DOI:** 10.1038/s41598-017-16336-y

**Published:** 2017-11-23

**Authors:** Jacqueline Farinha Shimizu, Caroline Sprengel Lima, Carina Machado Pereira, Cintia Bittar, Mariana Nogueira Batista, Ana Carolina Nazaré, Carlos Roberto Polaquini, Carsten Zothner, Mark Harris, Paula Rahal, Luis Octávio Regasini, Ana Carolina Gomes Jardim

**Affiliations:** 1 Genomics Study Laboratory, São Paulo State University, IBILCE, S. José do Rio Preto, SP Brazil; 2 Laboratory of Virology, Institute of Biomedical Science, ICBIM, Federal University of Uberlândia, Uberlândia, MG Brazil; 3 Laboratory of Green and Medicinal Chemistry, São Paulo State University, IBILCE, S. José do Rio Preto, SP Brazil; 4 School of Molecular and Cellular Biology, Faculty of Biological Sciences and Astbury Centre for Structural Molecular Biology, University of Leeds, Leeds, LS2 9JT United Kingdom

## Abstract

Hepatitis C virus (HCV) is one of the leading causes of liver diseases and transplantation worldwide. The current available therapy for HCV infection is based on interferon-α, ribavirin and the new direct-acting antivirals (DAAs), such as NS3 protease and NS5B polymerase inhibitors. However, the high costs of drug design, severe side effects and HCV resistance presented by the existing treatments demonstrate the need for developing more efficient anti-HCV agents. This study aimed to evaluate the antiviral effects of sorbifolin (**1**) and pedalitin (**2**), two flavonoids from *Pterogyne nitens* on the HCV replication cycle. These compounds were investigated for their anti-HCV activities using genotype 2a JFH-1 subgenomic replicons and infectious virus systems. Flavonoids **1** and **2** inhibited virus entry up to 45.0% and 78.7% respectively at non-cytotoxic concentrations. The mechanism of the flavonoid **2** block to virus entry was demonstrated to be by both the direct action on virus particles and the interference on the host cells. Alternatively, the flavonoid **1** activity was restricted to its virucidal effect. Additionally, no inhibitory effects on HCV replication and release were observed by treating cells with these flavonoids. These data are the first description of **1** and **2** possessing *in vitro* anti-HCV activity.

## Introduction

Hepatitis C virus (HCV) was identified in 1989 as the causative agent of hepatitis C^1^. It infects millions of people worldwide and is the major cause of liver disease and transplantation. According to World Health Organization (WHO), more than 350,000 people die currently from liver disease related to HCV infection^2^.

HCV is an enveloped, single stranded positive-sense RNA virus, which belongs to the Flaviviridae family, genus *Hepacivirus* ^3^. There is no effective vaccine for prevention of the HCV infection and, until recently, the only treatment for HCV infected patients was based on pegylated interferon and ribavirin association (PEG-IFN + RBV)^4^. The availability of new direct acting antivirals (DAAs) such as simeprevir, daclatasvir and sofosbuvir have increased rates of sustained virological response (SVR) with treatment efficacies as high as 90% for most common HCV genotypes^5–8^. However, the current treatments present several side effects, high costs^9^ and resistant variants were described even for the recent therapies approved by Food and Drugs Administration (FDA)^10,11^. Therefore, despite the introduction of interferon-free regimens, the therapy regime in many countries is still based on PEG-IFN + RBV.

The high costs and potential for developing viral resistance presented by the existing treatments demonstrate the need for improving therapeutic options against HCV. In this context, natural sources have demonstrated to provide a wide source of compounds, which can be evaluated for their antiviral properties^12^.

Flavonoids represent an important class of compounds, which are produced by plants as a response to microbial infections^13^. Several flavonoids have been described to possess antiviral activities. Epigallocatechin from green tea was demonstrated to inhibit replication of Enterovirus 71^14^, Chikungunya virus^15^ and HCV^16^. Naringenin from grapefruit showed antiviral effects against Herpes simplex virus type 1 (HSV-1)^17^ and HCV^18^. Silibin and ladanein have also demonstrated anti-HCV activities by inhibiting the viral entry step^19,20^. In this context, the high structural similarity between ladanein and flavonoids from *Pterogyne nitens* Tul. (Leguminosae) and other active flavonoids encouraged us to search for their potential anti-HCV activity by inhibition of viral entry.

*Pterogyne nitens*, popularly named, as “bálsamo”, “cocal”, “amendoim-bravo” and “yvi-raró”, is the unique member of its genus^21^. Ethnopharmacological studies in Guarani indigenous communities from Argentina indicated that cold aqueous preparations from its stem barks have been used for the treatment of helminthic infestations, mainly against *Ascaris lumbricoides* ^22^. Chemically, this species presents a variety of bioactive metabolites, including guanidine alkaloids^23^, triterpenes and steroids^24^. Phenolic compounds isolated from flowers showed myeloperoxidase inhibitory (MPO) and radical scavenging activities^25^. Studies with flavonoids from this plant have demonstrated their antiproliferative effect against melanoma cells^26^, inhibition of MPO^27,28^, radical scavenging properties and antioxidant activities^29–32^, as well as a potent antifungal activity against opportunistic fungi^33^.

Thus, the aim of this study was to investigate the effects of sorbifolin (**1**) and pedalitin (**2**) from *P. nitens* on HCV cycle. The data obtained displayed that these compounds strongly inhibited HCV entry to the hepatocytes by either the direct action on virus particles or the interference on the hepatocytes, but had no effect on virus replication and release.

## Results

### Anti-HCV activity of Sorbifolin and Pedalitin

**Figure 1:**
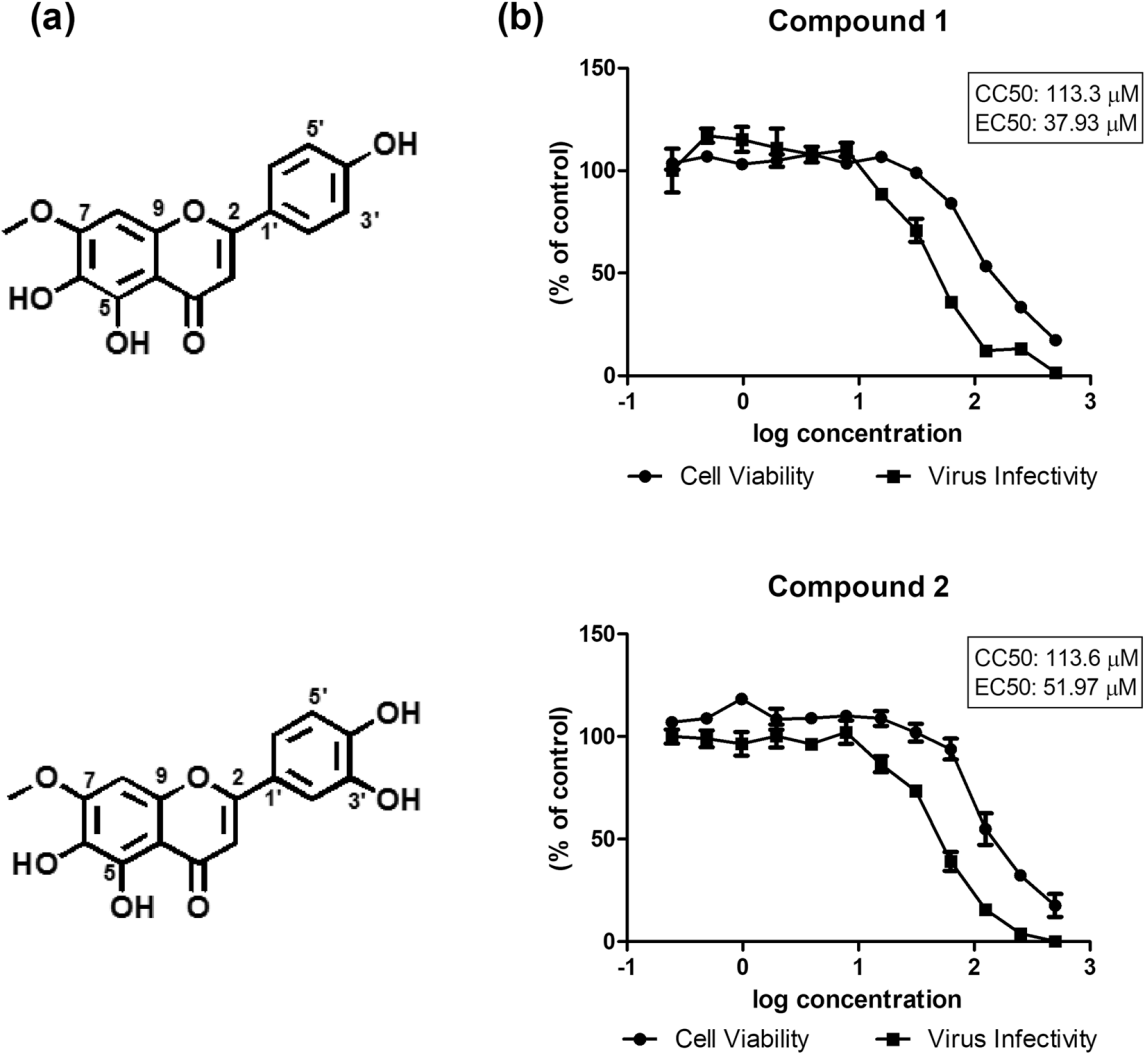
Flavonoids from *Pterogynes nitens*. The structure of sorbifolin (**1**) and pedalitin (**2**) (**a**). Cell viability and virus infectivity of flavonoids in näive Huh-7.5 cell line, cells were treated with compounds (**1**) and (**2**) at two-fold dilution 500 to3.9 µM for 72 h (**b**). DMSO 0.1% was used as non-treated control. Mean values of experiments are shown.

To evaluate the anti-HCV activity of the flavonoids **1** and **2** from *P. nitens* leaves (Fig. 1A), cytotoxicity screening of these compounds in näive Huh-7.5 cells was carried out. Cells were treated with increasing doses of each compound for 72 hours, and cell viability was measured by the MTT assay. The same assay was performed with Huh-7.5 cells infected with JFH-1 HCVcc and the antiviral activity of compounds was measured by luminescence levels. The results showed a tolerance of Huh-7.5 cell lines under treatment with both compounds which decreased HCV infectivity in a dose-dependent manner (Fig. 1B). The effective (EC_50_) and cytotoxic concentration (CC_50_) was determined for both compounds (37.93 and 113.3 µM for compound **1**; 51.97 and 113.6 µM compound **2**). Therefore, further assays were performed for a better understand of their antiviral activity.

We also evaluated the ability of the compounds to inhibit an alternative genotype of HCV.

**Figure 2:**
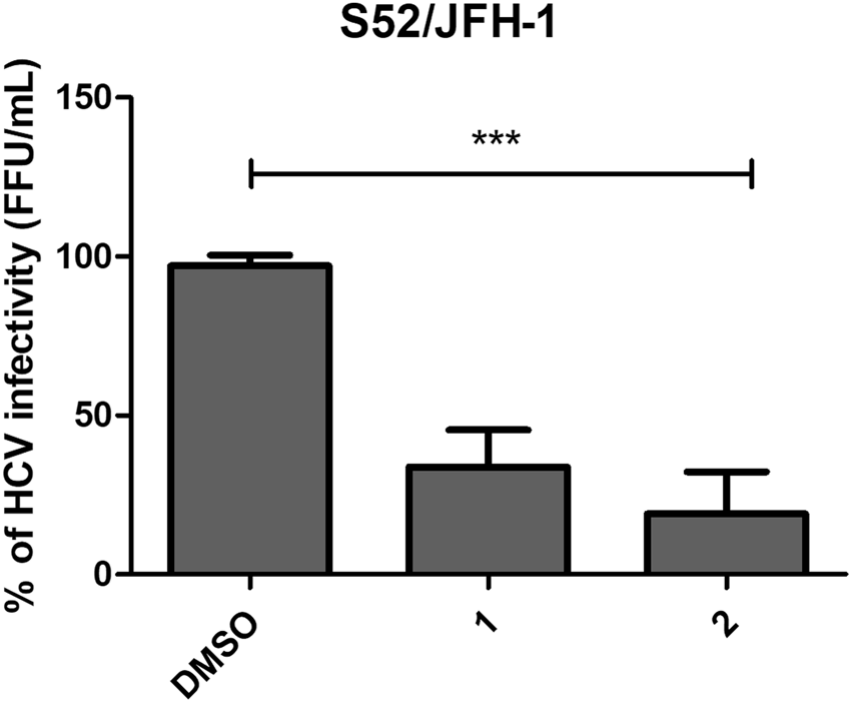
Inhibitory effect of compounds 1 and 2 against HCV genotype 3. Huh-7.5 cells were infected with S52/JFH-1 HCVcc and compounds at 50 μM concentration were immediately added to cells. The intracellular virus was titrated 72 h.p.i. by analyzing focus-forming units per milliliters (FFU/mL). DMSO 0.1% was used as non-treated control. P < 0.01 vs DMSO was considered significant.

To do this we used the full-length intergenotypic recombinant HCV genotype 3a/2a S52/JFH-1 replicon, which contains the core to NS2 protein regions from S52 replicon and the 5′UTR and NS3 protein to 3′UTR regions from JFH-1 replicon^34^. Cells were infected with HCVcc genotype 3a/2a S52/JFH-1 and immediately treated with each compound for 72 hours. Our results demonstrated that the compounds **1** and **2** also blocked the HCVcc S52/JFH-1 infectivity up to 81 and 66,25%, respectively (P < 0,001) (Fig. 2). These data corroborate with genotype 2a inhibitory rates, demonstrating the anti-HCV genotype-independent activity of these compounds.

### Sorbifolin and Pedalitin inhibit HCV entry to the host cells

**Figure 3:**
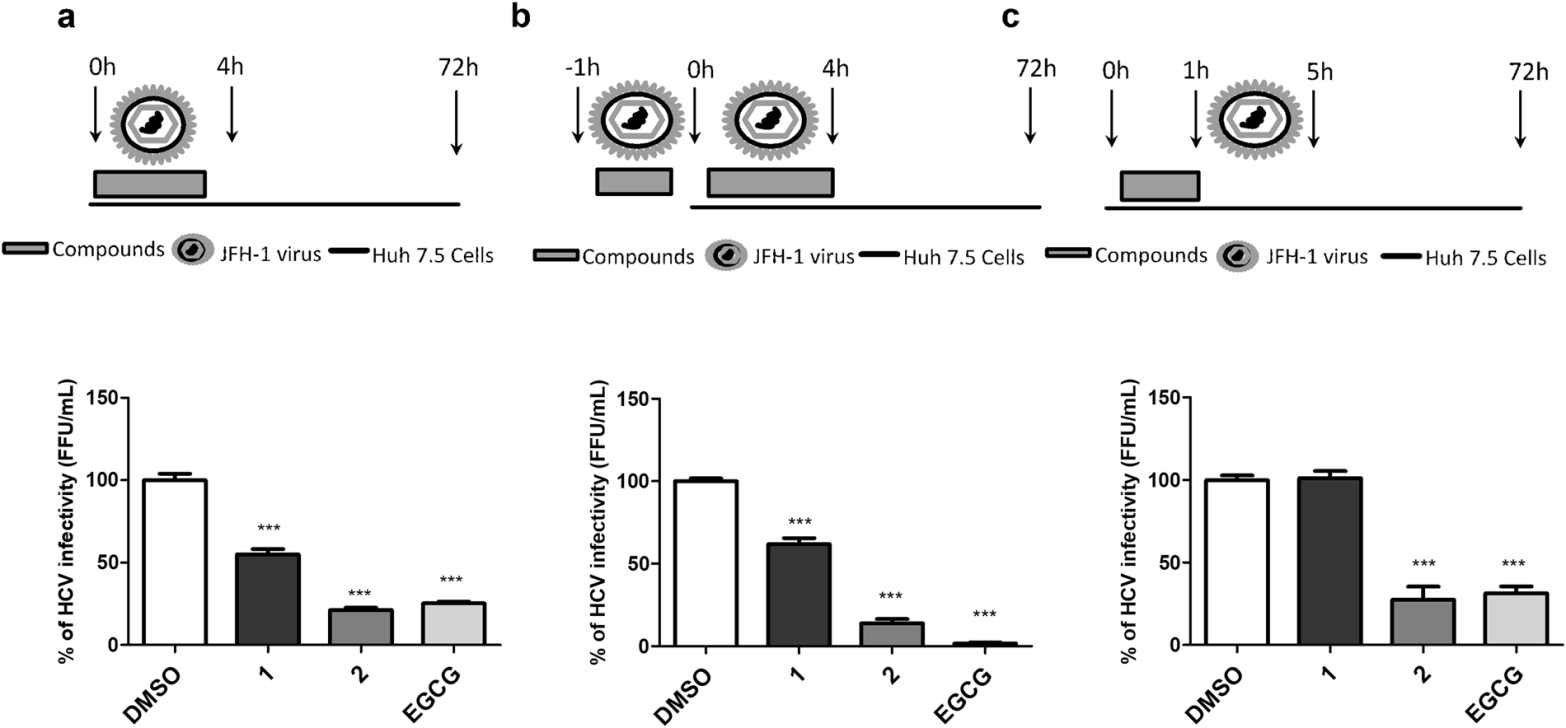
Effects of (**1**) and (**2**) on HCV infectivity. Infectious supernatant and compounds were added at different times to the cells, and the intracellular virus was titrated 72 h post-infection by analyzing focus-forming units per milliliters (FFU/mL). The percentage of infection was calculated using as reference the DMSO non-treated control. For entry assay, Huh-7.5 cells were infected with JFH-1 HCVcc and compounds 1 and 2 were immediately added. After 4 h, the supernatant was removed and replaced with fresh medium after repeated washes with PBS to remove the inoculum (**a**). For virucidal assay, JFH-1 HCVcc were incubated with (**1**) or (**2**) for 1 h prior to the infection. After that, the inoculum was used to infect naïve Huh-7.5 cells for 4 h. Cells were exhaustively washed and medium replaced (**b**). In the pre-treatment assay, cells were previously treated with compounds (**1**) and (**2**) for 1 h prior to the infection. Cells were washed to remove compounds and infected with JFH-1 virus for 4 h. Supernatants were removed, cells were washed to complete virus removal and were incubated with fresh media for up to 72 h post-infection (**c**). DMSO 0.1% was used as non-treated control and EGCG was used as control for entry blockade. Mean values of three independent experiments each measured in triplicate including the standard deviation are shown. P < 0.001 vs DMSO was considered significant.

To analyse the effects of compounds **1** and **2** on HCV entry to the host cells, virus-containing inoculum and compounds at 50 µM were simultaneously added to Huh7.5 cells and incubated for 4 hours. Cells were extensively washed with PBS to remove any remaining virus or compounds, and replaced with fresh medium for 72 hours. The intracellular virus was titrated by using a focus formation unit assay. The results demonstrated that **1** and **2** were able to block HCV entry up to 45.0%and 78.7%, respectively (Fig. 3A).

To further investigate the inhibitory activity of compounds **1** and **2** on virus entry, supernatant containing JFH-1 HCVcc was incubated with 50 μM of each compound for 1 hour at 37 °C prior to the infection of Huh-7.5 cells. The inoculum of virus and compound were transferred to the naïve cells and incubated for 4 hours. Cells were washed for the complete removal of the inoculum and replaced with fresh media for 72 hours when virus was titrated. A significant virucidal activity was observed for both compounds **1** and **2** which blocked 38.2% and 86% of virus entry, respectively (p < 0.001) (Fig. 3B). These data suggest that an anti-HCV mechanism of action for these compounds might be related to a direct action on the virus particle structure.

We also investigated whether the antiviral activity of these compounds was related to their effects on host cells. For this, Huh-7.5 cells were pre-treated with the compounds for **1** hour at 37 °C, followed by washes with PBS to remove any trace of compounds, prior to infection with JFH-1 HCVcc virus for 4 hours. Supernatant was replaced by fresh media after further PBS washes and intracellular virus was titrated at 72 hpi. The analysis showed that the flavonoid **2** significantly inhibited infectivity when cells were previously treated with this compound, blocking 72.5% of virus entry (Fig. 3C). On the other hand, compound **1** was not able to inhibit HCV entry by the prior treatment of cells. In addition to the above data, these results suggest that compound **2** seems to act on both the virus particle and the host cells.

### Sorbifolin and Pedalitin showed no effect on HCV replication or release

**Figure 4:**
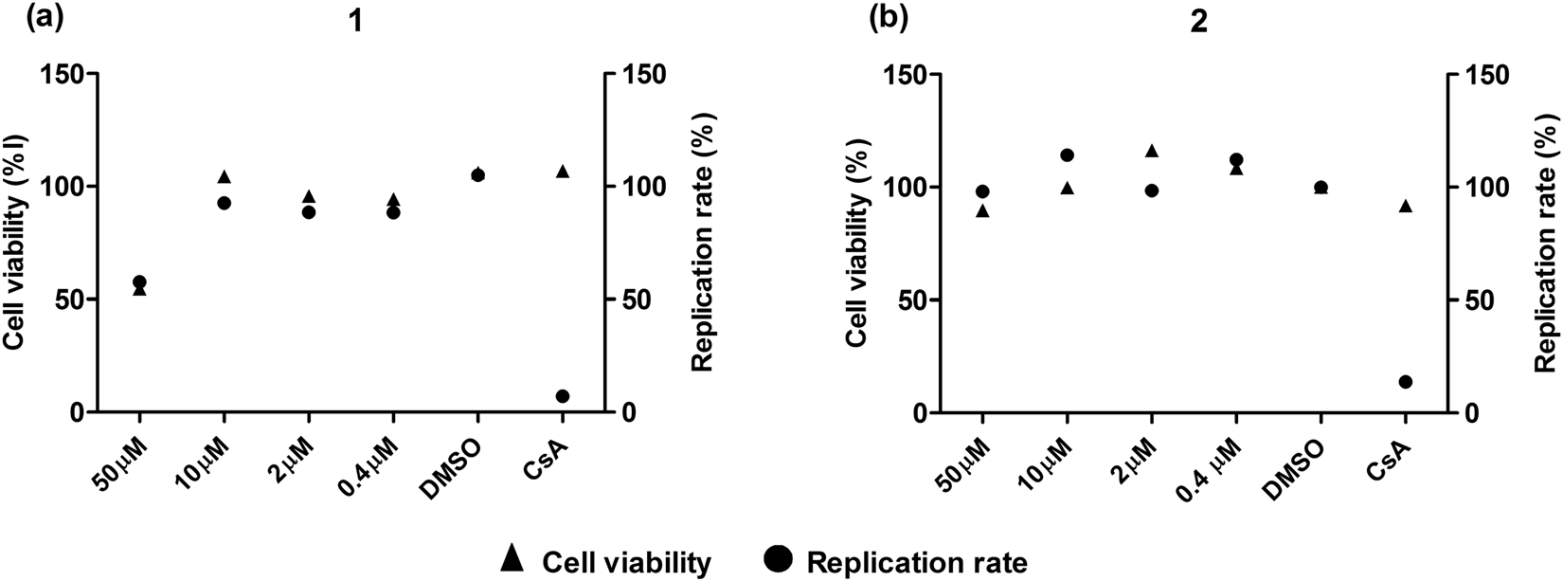
Effect on HCV replication. Huh-7.5 cells stably harboring subgenomic replicon SGR-FEO-JFH-1 were treated with (**1**) (**a**) and (**2**) (**b**) at 50, 10, 2 and 0.4 µM for 72 h. Cell viability (▴) and replication (●) were evaluated by MTT and luciferase assays, respectively. DMSO 0.1% was used as non-treated control and cyclosporine A (CsA) at 1 µM was used as control of inhibition of replication. Mean values of experiments are shown and P < 0.001 vs DMSO was considered significant.

Flavonoids were also described to inhibit other steps of HCV life cycle, for example the blockage of genome replication by apigenin^35^ and xanthohumol^36^, and the interference with virus release by naringenin^18,37^. The effects of the flavonoids **1** and **2** on HCV replication were evaluated using a firefly luciferase HCV subgenomic replication system (SGR-FEO-JFH-1)^38^. Huh7.5 cells stably harboring the SGR-FEO-JFH-1 were treated with varying concentrations of the compounds from 0.4–50 μM and incubated for 72 h to assess both the cytotoxicity and antiviral effects. Cell viability and HCV replication levels were measured by MTT and luciferase assays, respectively. The results showed that non-cytotoxic concentrations of **1** and **2** had no effect on HCV replication (Fig. 4).

**Figure 5:**
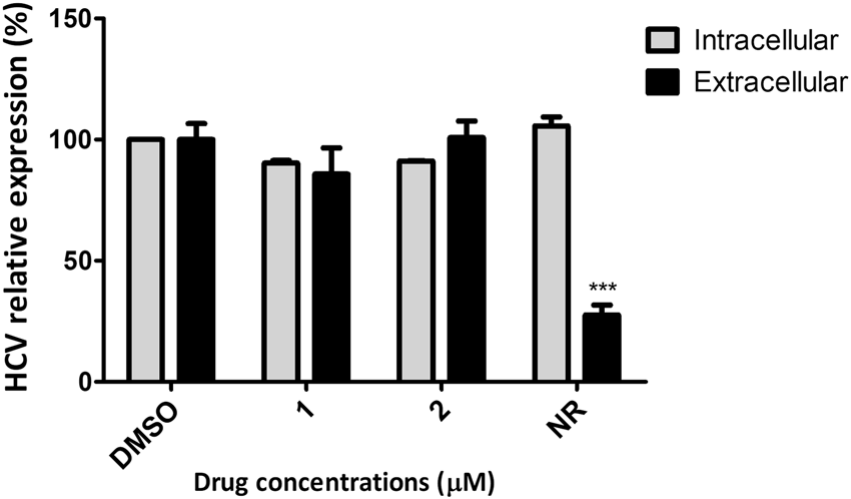
The capacity of compounds 1 and 2 to inhibit HCV release. Huh-7.5 cells previously infected with JFH-1 virus were plated 48 h prior treatment. Compounds (**1**) or (**2**) were added at 50 μM and incubated for 24 h. Supernatant was collected and cells were harvested, and intra and extracellular RNA were quantified by qPCR. DMSO 0.1% was used as non-treated control and naringenin (NR) at 400 μM was used as positive control of HCV secretion. Mean values of two independent experiments each measured in triplicate including the standard deviation are shown. ***P < 0.005 vs extracellular. ***P < 0.001 vs DMSO.

The effect of **1** and **2** on HCV release was evaluated by the quantification of intra and extra-cellular virus RNA using qPCR. Neither compound showed any inhibition of HCV release (Fig. 5) since there was no significant difference between intra and extracellular HCV RNA levels after 24 h of treatment.

### Sorbifolin and Pedalitin did not synergistically inhibit HCV when combined with Sofosbuvir

**Figure 6:**
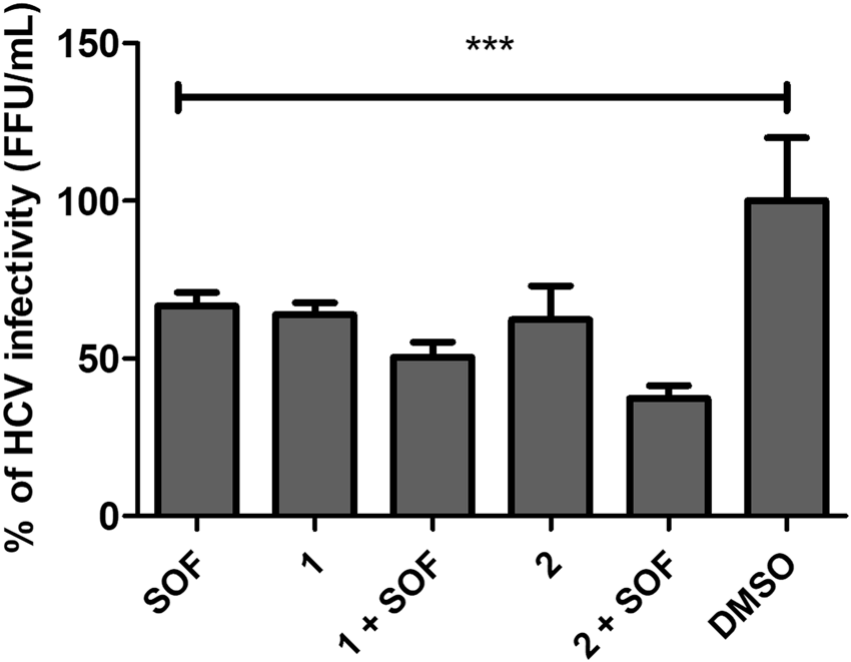
Effect of compounds 1 and 2 in monotherapy or combined with Sofosbuvir. Huh-7.5 cells were infected with JFH-1 HCVcc and compounds at specific concentration were immediately added to cells. After 72 h, cell viability (**a**) and virus infectivity (**b**) were measured. Compound 1, 2 and Sofosbuvir (SOF) were tested at 10 µM, 20 µM and 0.6 µM, respectively. DMSO was used as non-treated control. P < 0.01 vs DMSO was considered significant (***).

To evaluate whether compounds **1** and **2** enhance the anti-HCV activity of clinically used anti-HCV drugs. Huh-7.5 cells were infected with JFH-1 HCVcc and immediately treated with sub-EC_50_ concentrations of compounds **1** (10 µM), **2** (20 µM) and Sofosbuvir (0.6 µM) in monotherapy or in combined treatments. Our results showed that as monotherapy, compounds **1**, **2** and Sofosbuvir inhibited 36.2%, 38% and 33.4% of HCV, respectively. In combined treatment, compound **1** plus Sofosbuvir inhibited 49.6% and compound **2** plus Sofosbuvir inhibited 62.7% (Fig. 6). Although, a slight increase in HCV inhibition has been observed, no statistically significant differences were observed between combined treatments and the monotherapies (P < 0.001). All treatments were considered statistically different from the DMSO control (P < 0.001).

## Discussion

Our results demonstrated that the flavonoids Sorbifolin and Pedalitin isolated from Brazilian natural plants were able to block HCV entry to the host cells in a genotype independent manner. These data corroborate with previous findings concerning the activity of flavonoids on virus entry. In 2012, Calland *et al*. showed that the flavonoid EGCG was a potent HCV entry inhibitor^16^. More recently, delphinidin was identified as having potent activity against HCV entry, and it was shown that the mechanism of entry blockage observed for EGCG and delphinidin were the alteration in the structure of the HCV viral particles caused by these compounds, preventing the virus from binding to the cells^39^. Another mechanism of action was observed to silibinin, a flavonoid reported to block the HCV post-attachment step by altering clathrin mediated endocytosis and endosomal trafficking, resulting in a protective effect on host cells^20^. Anggakusuma *et al*. verified the anti-HCV activity of curcumin, a natural phenolic compound that inhibits HCV entry of all major genotypes by affecting virion envelope fluidity and by preventing HCV cell-to-cell transmission. By using quantum chemical and chemometric methods, Souza *et al*. demonstrated semi-empirically the predicted potential activity of three flavones on picornavirus particles^40^. Haid *et al*. isolated ladanein from *Marrubium peregrinum*, a flavone with potent and broad-spectrum antiviral activity against the entry of all HCV genotypes into human hepatocytes.

Erlejman *et al*. also demonstrated that the protective effects of flavonoids on cell membranes were mainly associated to a higher number of hydroxyl groups present in the molecules which is also related to their hydrophilicity and interactions between flavonoids and lipid bilayers^41^. Therefore, the biological activities of flavonoids were often associated with the types and number of functional radical groups in the chemical structure of different compounds^42,43^.

The structural difference between flavonoids **1** and **2** is a single radical, since flavonoid **2** the differences observed in the anti-HCV activity of flavonoids **1** and **2** could be explained.

The latest advance in therapy against HCV is the development of the DAAs, which target the replication step. As result, it succeeded in decreasing the viral replication and improved SVR rates^44^. However, due to an error-prone RNA polymerase and lack of proofreading in HCV^45^, as well the natural presence of virus quasispecies^46^, resistant variants to the current available HCV treatment have been reported^47,48^. Drug combination therapy is considered to be a promising approach to increase therapeutic efficacy and decrease drug resistance in comparison with mono-drug therapy^49^. However, compounds **1** and **2** did not synergistically inhibited HCV when combined with Sofosbuvir. Although these flavonoids did not enhance DAAs activity, new therapeutical approaches which target further stages of the HCV lifecycle cycle are necessary. These compounds were able to block viral entry and thus have demonstrated potent antiviral activity, making them an interesting target for future therapies against HCV since they could prevent virus infection at a very early stage in the virus replicative cycle.

In summary, the results showed that flavonoids from *P. nitens* inhibited HCV entry *in vitro*. Further analyses are necessary clarify the mechanisms of these flavonoids act against HCV infection. To the best of our knowledge, this is the first description of flavonoids from *P. nitens* leaves, which were screened against HCV *in vitro* and demonstrated antiviral effect by blocking entry stage of HCV cycle. The compounds **1** and **2** were previously documented to be isolated from other plant species, including *Ruellia tuberosa* ^50^, *Mentha pulegium*, and *M. suaveolens* ^51^. However, no antiviral activity was described. Therefore, these finds may be useful for the future development of new anti-HCV approaches.

## Methods

### Phytochemical Procedures

Leaves of P*. nitens* were collected in the Institute of Biosciences, Letters and Exact Sciences, São Paulo State University (UNESP), São José do Rio Preto, SP, Brazil (20°47′02.4′′S 49°21′36.0′′W) in July 2014. A voucher specimen (10291) was deposited in the Herbarium of Ilha Solteira (HISA) of the Faculty of Engineering, University of São Paulo State (Unesp), Ilha Solteira, SP, Brazil. The shade-dried leaves (630 g) was ground in a knife grinder and extracted twice (48 h, room temperature) by maceration with ethanol. The solvent was removed by filtration resulting in the ethanol extract. Ethanol extract (10 g) was submitted to reversed-phase C-18 silica gel CC (22 × 5 cm) eluted with ethanol: water gradient, affording 13 fractions of 300 mL each (L1‒L13), which were combined after comparison of their TLC profile [ethyl acetate: water: formic acid: acetic acid (100:27:11:11)] revealed with anisaldehyde-sulphuric acid reagent. Fraction L8 (412 mg) was subjected to gel permeation column chromatography using Sephadex® LH-20 (100 × 3 cm), eluted with ethanol to afford 60 subfractions of 30 mL each (SL1‒SL60). All the subfractions were compared by TLC profile using [ethyl acetate: water: formic acid: acetic acid (100:27:11:11)] revealed with anisaldehyde-sulphuric acid reagent. Subfractions SL31‒SL36 (50 mg) and SL44‒SL53 (70 mg) presented one spot on TLC plates, suggesting purification of compounds **1** and **2**, respectively. Subfractions SL37‒SL43 demonstrated mixture of **1** and **2**. Structures of compounds **1** and **2** were identified by comparison with literature data, mainly ^1^H and ^13^C NMR values^52–54^. The MR spectra were obtained in DMSO-*d*_6_ solution, using a Varian INOVA 500 spectrometer (11.7 T), operating at 500 MHz for ^1^H and 125 MHz for ^13^C. Sorbifolin (**1**): TLC Rf 0.57 [ethyl acetate: water: formic acid: acetic acid (100:27:11:11)], ^1^H NMR *δ*_H_ (multiplicity; *J* in Hz; position): 12.5 (br s 5-OH), 3.87 (s; 7-OCH3), 7.89 (d; 8.5; H-2′ and H-6′), 6.90 (d; 8.5; H-3′ and H-5′), 6.85 (s; H-8), 6.70 (s; H-3). ^13^C NMR *δ*c (position): 182.5 (C-4), 154.7 (C-7), 150.1 (C-9), 164.3 (C-2), 161.4 (C-4′), 116.4 (C-3′ and C-5′), 102.8 (C-3), 121.7 (C-1′), 105.3 (C-10), 130.2 (C-6), 91.5 (C-8), 128.8 (C-2′ and C-6′), 146.4 (C-5). Pedalitin (**1**): TLC Rf 0.34 [ethyl acetate: water: formic acid: acetic acid (100:27:11:11)], ^1^H NMR *δ*_H_ (multiplicity; *J* in Hz; position): 12.6 (br s; 5-OH), 3.92 (s; 7-OCH_3_), 7.44 (d; 1.5; H-2′), 7.43 (dd; 1.5 and 8.5; H-6′), 6.90 (d; 8.5; H-5′), 6.85 (s; H-8), 6.68 (s; H-3). ^13^C NMR *δ*c (position): 182.0 (C-4), 154.3 (C-7), 149.6 (C-9), 163.9 (C-2), 149.6 (C-4′), 145.7 (C-3′), 115.9 (C-3′), 102.5 (C-3), 121.7 (C-1′), 105.0 (C-10), 129.9 (C-6), 91.0 (C-8), 113.4 (C-2′) and 118.8 (C-6′).

### Partition coefficient (n-octanol/water) measured by HPLC method

The partition coefficient was calculated using the HPLC method according procedures described by OECD Guidelines for the Testing of Chemicals^52^. The equipment used was a Shimadzu HPLC model CBM 20-A (Shimadzu®) equipped with photodiode array detector (model SPD-M20A), binary pumping system mobile phase (model LC-20AD_XR_), solvent degasser (model DGU-20A_3R_), injector system (model SIL-20AC) and a Gemini C-18 chromatographic column (Phenomenex®, 250 mm × 4.6 mm, 5 μm, 100 Å). For HPLC method was used an isocratic mode [methanol:water (3:1)] and 1.0 mL/min. The sample volume injected was 20 µL and the wavelength was 354 nm. The following substances were used as references to construct the curve log *K* × log *P*: thiourea, aniline, phenol, acetophenone and benzoic acid. The capacity factor (log *K*) of the compounds was determined from their retention times and interpolated in linearity curve log *K* × log *P*. The partition coefficient (Log P_o/w_) of flavonoids **1** and **2** was 1.6 and 1.3, respectivily (Figure S1).

### Sample Preparation

The lyophilized compounds were dissolved in dimethyl sulfoxide (DMSO) in stock solutions and stored at −80 °C. Compounds were diluted in complete medium immediately prior to the experiments to reach a maximum final concentration of 0.1% DMSO. For all the assays performed, non-treated control was added of DMSO at the same final concentration. Epigallocatechin-gallate (EGCG, Sigma-Aldrich) was used as positive control for entry, pre-treatment and virucidal assays^15,16,55^. Cyclosporine A (CsA) was used as positive control of inhibition of replication^56,57^.

### HCV Replicons

Antiviral activity of compounds was evaluated by using both subgenomic and full-length HCV replicon systems. The full-length HCV genotype 2a JFH-1^58^ and intergenotypic recombinant HCV genotype 3a/2a S52/JFH-1^59^ replicons were used to perform virus assays as described below. The subgenomic replicon SGR-FEO-JFH-1, based on the non-structural proteins NS3-NS5B of HCV genotype 2a JFH-1 strain inserted of a firefly luciferase-neomycin phosphotransferase fusion protein, was used to performed replication assay^38^.

### Cell Culture

The human hepatocyte cell line Huh-7.5 (Apath LLC, Brooklyn, NY) was maintained in Dulbecco’s modified Eagle’s medium (DMEM; Sigma–Aldrich) supplemented with 100 U/mL penicillin (Gibco Life Technologies), 100 mg/mL streptomycin (Gibco Life Technologies), 1% non-essential amino acids (Gibco Life Technologies), 1% HEPES (Gibco Life Technologies, USA) and 10% fetal bovine serum (FBS; Cultilab) at 37 °C in a humidified 5% CO_2_ incubator. Huh-7.5 cells stably harboring the SGR-FEO-JFH-1 were cultured under the same condition, added of 500 µg/mL of G418 (Sigma-Aldrich, USA).

### Cell Viability

Cell viability was measured by MTT [3-(4,5-dimethylthiazol-2-yl)-2,5-diphenyl tetrazolium bromide] (Sigma–Aldrich) assay. Huh-7.5 or SGR-FEO-JFH-1 Huh-7.5 cells were seed in 96-well microplates and incubated at 37 °C in a humidified 5% CO_2_ incubator overnight. Drug-containing medium at different concentrations was added to the cell culture. After incubation for 72 hours, DMEM containing MTT at1 mg/mL was added to each well, incubated for 1 hour and replaced with 100 μL of DMSO to solubilize the formazan crystals. The absorbance was measured at 562 nm on a plate reader (FLUOstar Omega/BMG LABTECH, Offenburg, BW, DE). Cell viability was calculated according to the equation (T/C) × 100%, which T and C represented the mean optical density of the treated and control groups, respectively. DMSO was used as non-treated control. The cytotoxic concentration of 50% (CC_50_) was calculated using Prism (Graph Pad).

### Luciferase-based Replication

Huh-7.5 cells stably harboring the SGR-FEO-JFH-1 were seeded on 96-well plates at a density of 5 × 10^3^ per well and compounds at 50 µM, 10 µM, 2 µM and 0.4 µM were added. After 72 hours, cells were harvested by lysis with Passive Lysis Buffer (Promega) and HCV RNA replication was quantified by measuring luminescence levels using the Luciferase Assay System (Promega) and a plate reader (FLUOstar Omega/BMG LABTECH, Offenburg, BW, DE).

### Virus assays

HCVcc particles were generated as described previously^60^. Briefly, 8 × 10^6^ Huh-7.5 cells were electroporated with 10 μg of JFH-1^58^ or S52/JFH-1 RNA^59^ and infectious supernatant was collected from after 48 hours incubation. Naïve Huh7.5 cells were seeded the day before the assay was carried out. Cells were infected with JFH1cc virus^58^ at a multiplicity of infection (MOI) of 0.4 and compounds at specific concentrations were added at different times, depending on the stage of HCV cycle to be evaluated. The effective concentration of 50% (EC_50_) was calculated using Prism (Graph Pad).

### Inhibitory Effects on Entry Steps

For virus entry experiments, JFH-1 HCVcc was used to infect Huh-7.5 cells in complete medium with compounds for 4 hours. The supernatant plus virus was removed, cells were washed three times with PBS to completely remove the inoculum, replaced with fresh complete medium and incubated at 37 °C in a humidified 5% CO_2_ incubator. After 72 hours, the supernatant was removed, cells were fixed and intracellular virus was titrated.

### Pre-treatment assay

Huh-7.5 cells were pre-treated with each compound for 1 hour at 37 °C in a humidified 5% CO_2_ incubator prior to the infection. After incubation, cells were washed extensively to remove compounds and were infected with HCVcc JFH-1 virus for 4 hours. Infectious supernatant was removed, additional washes were performed to virus removal and fresh media was added. The virus was titrated 72 hours post-infection (hpi).

### Virucidal assay

For virucidal assay, infectious supernatant containing HCVcc JFH-1 virus was incubated with each compound for 1 hour at 37 °C prior to the infection. Then, the mixture was used to infect naive Huh-7.5 cells for 4 hours at 37 °C in a humidified 5% CO_2_ incubator. The inoculums were removed, cells were washed three times with PBS, replaced by fresh media and incubated 72 hours at 37 °C in a humidified 5% CO_2_ incubator. Virus was titrated 72 hpi.

### Virus Titration

Cells were fixed with 4% para-formaldehyde (PFA) 72 hpi, washed with 100 mM Glycine (Applichem), semi-permeabilized with 0.1% Triton X-100 (Vetec Labs) and stained for NS5A using sheep anti-NS5A^61^ and Alexa Fluor anti-sheep 594 secondary antibody. Infectivity was expressed as focus-forming units per milliliter of supernatant (FFU/mL).

### HCV Release/Assembly Assessment

To analyze the compounds **1** and **2** influence on HCV secretion, 2 × 10^5^ JFH-1 infected cells were seeded 48 hours before treatment. Then, the medium was replaced by fresh medium supplemented with compounds at 50 μg/mL as described by Nahmias *et al*.^37^. DMSO 0,1% was used as non-treated control and Naringenin (NR) at 400 μM was used as control of HCV secretion inhibition^37^. After 24 hours of incubation, RNA was extracted from the supernatant and from the cells using TRIzol reagent (Life Technologies, Carlsbad, CA, USA), and cDNA was synthesized with High-Capacity cDNA Archive (Applied Biosystems, Foster City, CA, USA). HCV expression analysis was performed by TaqMan Universal PCR Master Mix no AmpErase UNG (Applied Biosystems, Branchburg, NJ, USA) detecting the amplification of the HCV 5′UTR region (Forward: CGGGAGAGCCATAGTGG; Reverse: AGTACCAACAAGGCCTTTCG). The samples quality and normalization of levels of expression were obtained by amplification of the endogenous gene GAPDH. JFH1 release inhibition was calculated as a percentage of negative control.

### Combined Treatment with Sofosbuvir

Huh-7.5 cells were infected with JFH-1 HCVcc and treated immediately with compound 1 (10 µM), 2 (20 µM) and Sofosbuvir (0.6 µM) (SOVALDI®) in monotherapy or combined treatments. After 72 hours, cell viability and infection levels were measured. DMSO was used as non-treated control.

### Statistical Analysis

Individual experiments were performed in triplicate and all assays were performed a minimum of three times in order to confirm the reproducibility of the results. Differences between means of readings were compared using analysis of variance (One-way or Two-way ANOVA) and Students t test. P values of less than 0.05 (indicated by asterisks) were considered statistically significant.

## Electronic supplementary material

Supplementary Information
