# Supplementary material for: Flavonoids from Pterogyne nitens Inhibit Hepatitis C Virus Entry
Source: Sci Rep. 2017 Nov 23;7:16127. doi: 10.1038/s41598-017-16336-y (PMC5701011; doi:10.1038/s41598-017-16336-y)
Supplement: Supplementary file 1 — Supplementary Information [file 41598_2017_16336_MOESM1_ESM.docx]

**Flavonoids from *Pterogyne nitens* Inhibit Hepatitis C Virus Entry**

**Jacqueline Farinha Shimizu**^†§║^**,** **Caroline Sprengel Lima**^‡║^, Carina Machado Pereira^†^, Cintia Bittar^†^, Mariana Nogueira Batista^†^, Ana Carolina Nazaré^‡^, Carlos Roberto Polaquini^‡^, Carsten Zothner^⊥^, Mark Harris^⊥^, Paula Rahal^†^, Luis Octávio Regasini^‡^,Ana Carolina Gomes Jardim*^§†^

^†^Genomics Study Laboratory, São Paulo State University, IBILCE, S. José do Rio Preto, SP, Brazil

^§^Laboratory of Virology, Institute of Biomedical Science, ICBIM, Federal University of Uberlândia, Uberlândia, MG, Brazil

^‡^ Laboratory of Green and Medicinal Chemistry, São Paulo State University, IBILCE, S. José do Rio Preto, SP, Brazil

^⊥^School of Molecular and Cellular Biology, Faculty of Biological Sciences and Astbury Centre for Structural Molecular Biology, University of Leeds, Leeds LS2 9JT, United Kingdom

^║^These authors contributed equally to this work

* **Corresponding Author:** Tel. +55 34 3225-8679. Fax: +55 34 3225 8671. E-mail: jardim@ufu.br

**Supplementary Data**

**Figure S1.** Retention time of sorbifolin (**1**) from HPLC-DAD analysis.

0,0

2,5

5,0

7,5

10,0

12,5

15,0

17,5

min

0

5

10

15

20

25

mAU

354nm,4nm

2,893

4,010

**Chromatographic conditions:** MeOH:H_2_O (3:1), 354 nm and 1.0 mL/min.

**Figure S2.** Peak area of sorbifolin (**1**) from HPLC-DAD analysis.

0,0

2,5

5,0

7,5

10,0

12,5

15,0

17,5

min

0

5

10

15

20

25

mAU

354nm,4nm

3,994

96,006

**Chromatographic conditions**: MeOH:H_2_O (3:1), 354 nm and 1.0 mL/min.

**Figure S3.** Retention time of pedalitin (**2**) from HPLC-DAD analysis.

0,0

2,5

5,0

7,5

10,0

12,5

15,0

17,5

min

0,0

2,5

5,0

7,5

10,0

12,5

15,0

mAU

354nm,4nm

2,883

3,597

**Chromatographic conditions**: MeOH:H_2_O (3:1), 354 nm and 1.0 mL/min.

**Figure S4.** Peak area of pedalitin (**2**) from HPLC-DAD analysis.

2,5

5,0

7,5

10,0

12,5

15,0

17,5

min

0,0

2,5

5,0

7,5

10,0

12,5

mAU

354nm,4nm

2,389

97,611

**Chromatographic conditions**: MeOH:H_2_O (3:1), 354 nm and 1.0 mL/min.
